# Ruptured Abdominal Aortic Aneurysm Masquerading as Acute Appendicitis

**DOI:** 10.7759/cureus.95582

**Published:** 2025-10-28

**Authors:** Dimitrios A Chatzelas, Stefanos K Atmatzidis, Maria D Velikoudi, Vasileios Papaziogas

**Affiliations:** 1 2nd Department of Surgery, Faculty of Medicine, Aristotle University of Thessaloniki, “G. Gennimatas” General Hospital of Thessaloniki, Thessaloniki, GRC

**Keywords:** abdominal aortic aneurysm, acute appendicitis, differential diagnosis, endovascular repair, rupture

## Abstract

Ruptured abdominal aortic aneurysm (AAA) is a life-threatening emergency with high mortality. Its presentation is often atypical and may mimic more common abdominal conditions. We report the case of a 65-year-old man who presented with acute right lower quadrant pain initially suggestive of acute appendicitis. However, computed tomography (CT) revealed a large infrarenal AAA with a contained rupture. This atypical presentation highlights the importance of including AAA in the differential diagnosis of elderly patients with abdominal pain. Early imaging, particularly CT, is essential to differentiate AAA from other causes of acute abdomen and to prevent diagnostic delays. Maintaining a high index of suspicion is critical for timely recognition and improved outcomes.

## Introduction

Abdominal aortic aneurysm (AAA) is defined as a permanent, focal dilatation of the abdominal aorta exceeding at least 50% of its normal diameter, adjusted for age, sex, and race [1]. Although most AAAs remain asymptomatic and are detected incidentally, rupture represents the most catastrophic complication, with mortality rates of 65%-80%, even among patients who reach hospital care [1]. The classic triad of rupture, acute abdominal or back pain, hypotension, and a pulsatile abdominal mass, is observed in fewer than 50% of cases [2]. More often, patients present with nonspecific or misleading symptoms, leading to misdiagnoses such as appendicitis, diverticulitis, renal colic, or perforated viscus [2,3].

We report the case of a 65-year-old man who presented to the emergency department with acute abdominal pain initially suggestive of appendicitis, but subsequent imaging revealed a large, ruptured AAA. This report aims to raise awareness among emergency physicians about the atypical presentations of ruptured or symptomatic AAA, emphasizing the diagnostic challenges and the importance of maintaining a high index of suspicion to prevent potentially fatal delays in diagnosis.

## Case presentation

A 65-year-old man presented to the emergency department with acute-onset abdominal pain that began approximately six hours prior to admission. Initially epigastric, the pain quickly migrated to the right lower quadrant (RLQ). It was continuous, moderate to severe in intensity, and exacerbated by movement, coughing, or walking. The patient reported a single episode of vomiting but denied other gastrointestinal or urinary symptoms. He had a 23 pack-year smoking history and a medical history of arterial hypertension and dyslipidemia, managed with an angiotensin-converting enzyme inhibitor and a statin. On examination, he was fully conscious and oriented. Vital signs were stable: blood pressure 102/61 mmHg, heart rate 92 bpm, temperature 36.4°C, and oxygen saturation 98% on room air. Abdominal examination revealed mild distension and localized tenderness in the RLQ with a positive McBurney sign. Mild rebound tenderness was present, without guarding or rigidity. Bowel sounds were normal on auscultation. There was no history of constipation, diarrhea, or dysuria, and digital rectal examination was unremarkable. Laboratory results are summarized in Table 1.

**Table 1:** Blood test results at presentation WBC: white blood count, NE: neutrophils, RBC: red blood count, PLT: platelets, PT: prothrombin time, INR: international normalized ratio, aPTT: activated partial thromboplastin time, SGOT: serum glutamic-oxaloacetic transaminase, SGPT: serum glutamic-pyruvic transaminase, LDH: lactate dehydrogenase, CPK: creatine phosphokinase, CRP: C-reactive protein.

Blood test	Result	Reference range
WBC (×10^3^/μL)	18.2	4.3-10.3
NE (%)	81.4	41-73
RBC (×10^6^/μL)	3.91	4.38-5.77
Hematocrit (%)	38.2	39.5-51
Hemoglobin (gr/dl)	12.5	13.6-17.2
PLT (×10^3^/μL)	298	140-440
PT (sec)	11.7	10-15
INR	1.0	-
aPTT (sec)	23.7	26-36
SGOT (IU/L)	12	<40
SGPT (IU/L)	18	<41
Urea (mg/dL)	36	0-50
Creatinine (mg/dL)	1.02	0.7-1.2
LDH (IU/L)	139	135-225
CPK (IU/L)	114	38-190
CRP (mg/dL)	6.1	<0.5

**Figure 1:**
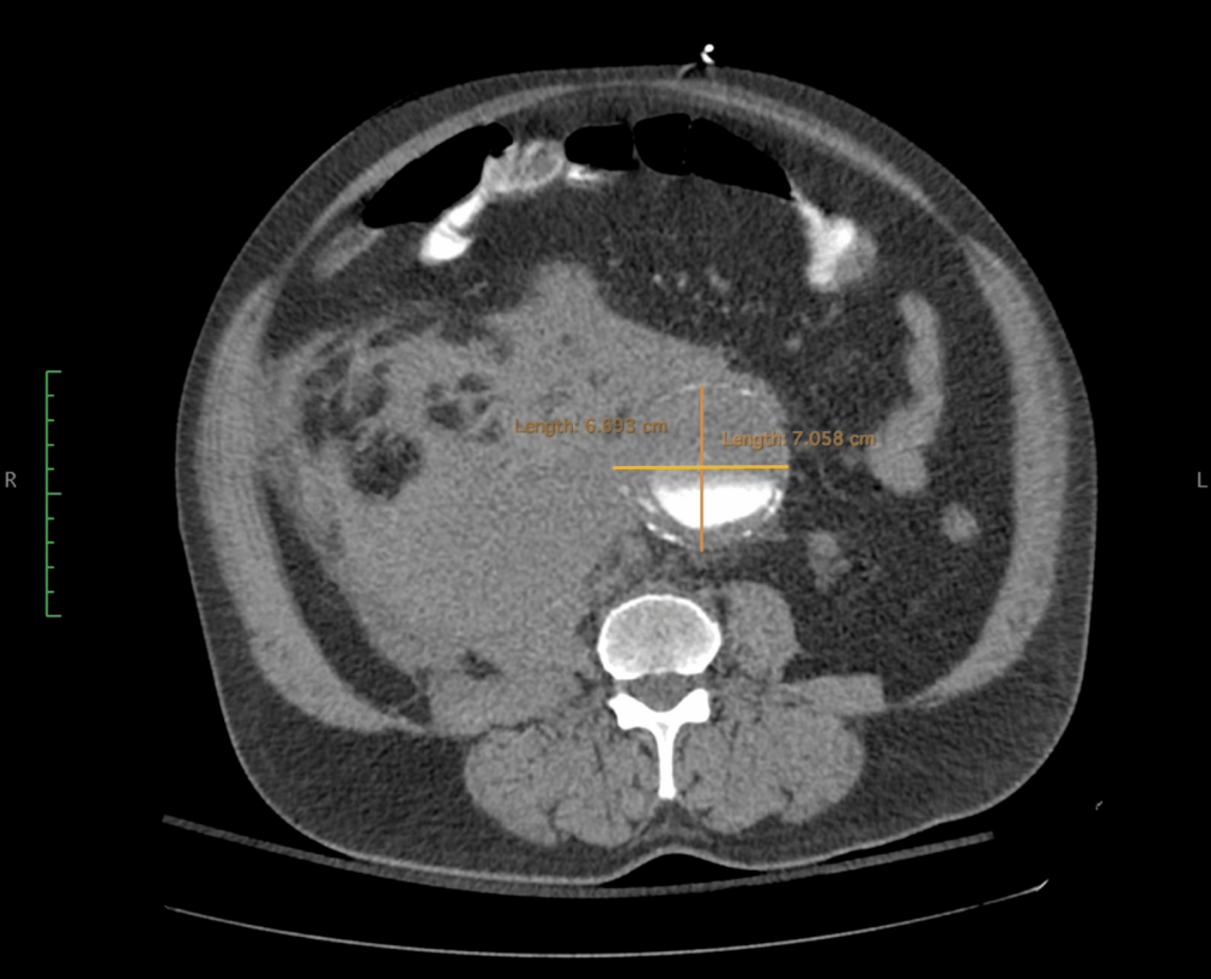
Computed tomography angiography showing an infrarenal abdominal aortic aneurysm measuring 70 mm in maximum diameter, with a contained rupture and associated retroperitoneal hematoma (estimated volume: 760 mL)

Based on the patient’s history, clinical findings, and laboratory results, the Alvarado score was calculated at 9/10, suggesting a high probability of acute appendicitis. Consequently, a contrast-enhanced CT scan of the abdomen and pelvis was performed following intravenous iodine and oral gastrografin administration. Unexpectedly, the CT revealed a previously undiagnosed infrarenal AAA measuring 70 mm in maximum transverse diameter, with evidence of a contained retroperitoneal rupture and a large associated hematoma (Figure 1). The proximal aortic neck measured 28 mm in diameter and 19 mm in length, with an angulation of approximately 45°. Both iliac arteries and access vessels were patent and demonstrated favorable anatomy and wall quality.

**Figure 2:**
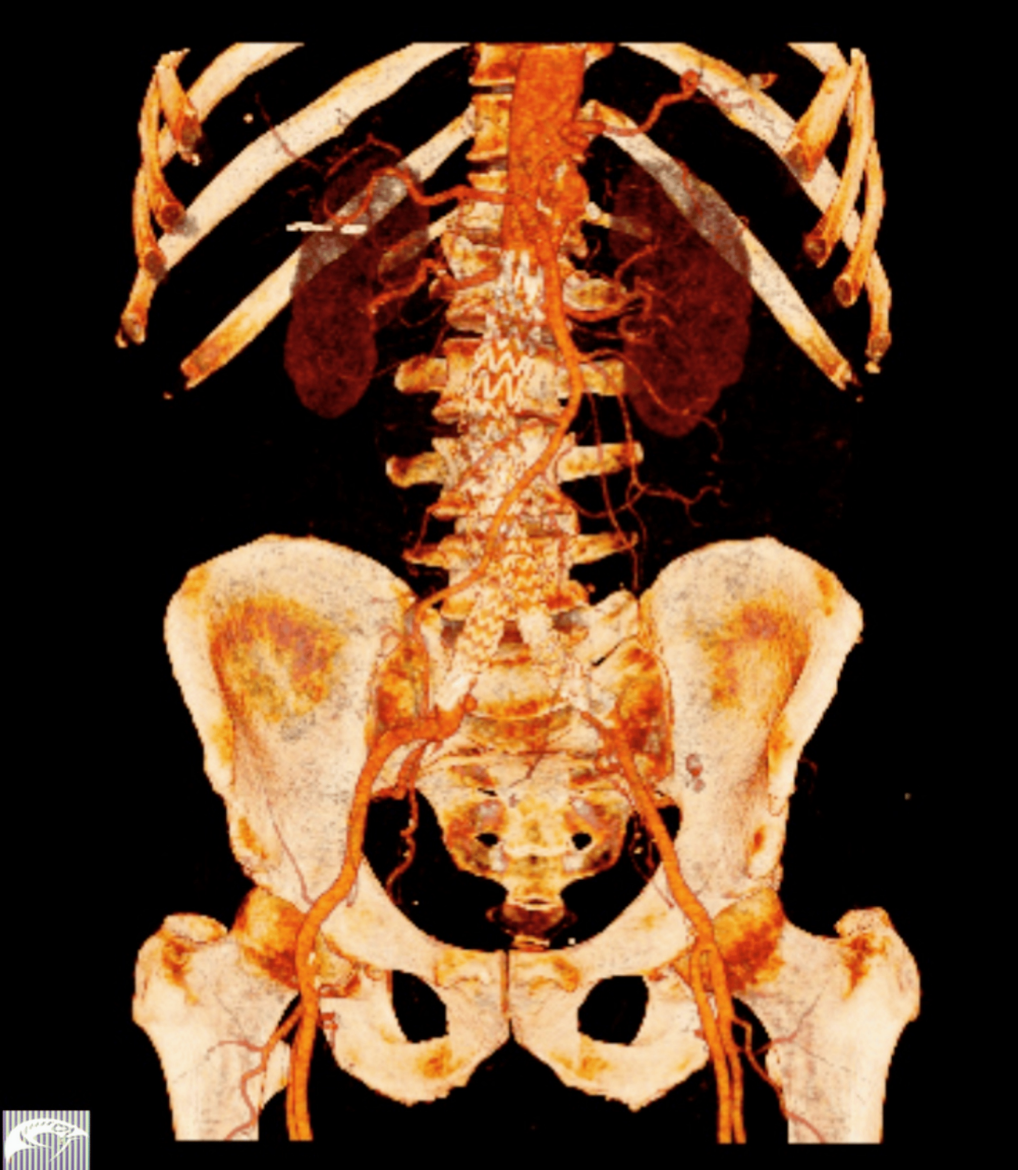
Postoperative computed tomography angiography showing the bifurcated abdominal aortic stent-graft in place, without evidence of endoleak

Immediately, the vascular surgery team was consulted. After obtaining informed consent, the patient was transferred to the hybrid operating room. Under local anesthesia and bilateral femoral cut-down, he underwent endovascular AAA repair (EVAR) using a 36-mm bifurcated self-expanding abdominal aortic Ankura™ stent-graft (Lifetech Scientific, Shenzhen, China), extending from just below the renal arteries to the iliac bifurcations. The procedure lasted approximately 90 minutes and was completed without intraoperative complications. The patient received two units of packed red blood cells and two units of fresh frozen plasma. Postoperatively, he was monitored in the high-dependency unit for 24 hours before transfer to the general ward. His recovery was uneventful, and he was discharged on postoperative day 3 under single antiplatelet therapy. Follow-up CT angiography (CTA) at one month showed no evidence of endoleak (Figure 2). At six months, the patient remained asymptomatic and continued regular clinical and imaging follow-up.

## Discussion

AAA is a prevalent degenerative vascular disease among elderly men, with smoking, hypertension, and dyslipidemia being the strongest risk factors [1]. Its prevalence in men over 65 years ranges from 4% to 8%, and rupture represents the most catastrophic complication. While elective AAA repair carries a relatively low perioperative mortality of 2%-5%, rupture is associated with mortality rates of 65%-80%, even among those who reach the hospital and undergo treatment [1].

The clinical presentation of ruptured AAA is often variable and misleading [2]. Reported manifestations range from asymptomatic cases to sudden death [2]. The classic triad of abdominal or back pain, hypotension, and a pulsatile abdominal mass is present in fewer than half of patients [2]. More commonly, elderly individuals present with vague or localized abdominal pain that mimics other acute surgical conditions such as appendicitis, diverticulitis, or renal colic [3-5]. In this case, the patient’s pain migrated from the epigastrium to the RLQ, closely resembling the classical progression of acute appendicitis [6]. Physical examination also revealed localized RLQ tenderness with a positive McBurney sign and mild rebound tenderness, findings typically suggestive of appendicitis [6]. His hemodynamic stability, absence of a palpable pulsatile mass, and normal inflammatory markers further reinforced this misleading clinical picture. The contained retroperitoneal hematoma extended along the right psoas muscle and iliac fascia, producing irritation of the somatic nerves of the lumbar plexus, which supply sensation to the RLQ and groin [3]. This neural irritation results in referred somatic pain, often simulating appendicitis or ureteric colic, even when the underlying pathology is retroperitoneal bleeding from an AAA [3,4].

Misdiagnosis of ruptured AAA is a well-recognized problem [3]. A recent meta-analysis reported that up to 42% of patients with ruptured AAA are initially treated for other intra-abdominal conditions before the vascular etiology is identified [7]. The diagnostic challenge underscores the need for a high index of clinical suspicion, as any delay in recognition and intervention significantly increases morbidity and mortality [7]. Our case highlights the importance of considering AAA in the differential diagnosis of elderly patients presenting with RLQ pain, particularly in those with vascular risk factors such as smoking and hypertension. RLQ pain should raise suspicion for appendicitis, but it is not pathognomonic, even when accompanied by localized tenderness [6]. The presence of back pain, unexplained hypotension, anemia, or atypical abdominal findings should prompt reconsideration of the diagnosis [6,7].

Imaging plays a decisive role in the differential diagnosis [1]. Ultrasound is a useful first-line modality, capable of confirming the presence of AAA with a sensitivity exceeding 95% for aneurysms larger than 50 mm [8]. However, its diagnostic accuracy may be limited in unstable patients or when attention is focused on the right iliac fossa rather than the aorta [8]. CTA remains the gold standard for diagnosing ruptured AAA [9,10]. It provides essential details on aneurysm morphology, the extent of retroperitoneal hematoma, and signs of active bleeding, while simultaneously excluding alternative diagnoses such as appendicitis or diverticulitis [10]. In this case, CTA not only established the correct diagnosis but also guided treatment planning by revealing favorable anatomy for endovascular repair, which is now considered the gold standard for ruptured AAA management [1].

## Conclusions

This case highlights the importance of maintaining a broad differential diagnosis in elderly patients presenting with abdominal pain. While appendicitis is a common consideration, especially when pain localizes to the right lower quadrant, clinicians must keep a high index of suspicion for AAA, as misdiagnosis or diagnostic delay can be fatal. Prompt CT imaging in ambiguous cases is essential to achieve diagnostic certainty and enable early referral to vascular surgery, directly influencing patient survival outcomes.
